# Therapeutic Efficacy of Aerobic Exercise Training along with Oak Husk Hydroalcoholic Extract for Amelioration of Inflammation in Obese Elderly Male Mice

**DOI:** 10.1155/2021/5585077

**Published:** 2021-05-03

**Authors:** Iman Zakavi, Shila Nayebifar, Elham Ghasemi, Aliasghar Valipour

**Affiliations:** ^1^Department of Public Health, Abadan Faculty of Medical Sciences, Abadan University of Medical Sciences, Abadan, Iran; ^2^Department of Sport Sciences, Faculty of Educational Sciences and Psychology, University of Sistan and Baluchestan, Zahedan, Iran; ^3^Department of Sport Sciences, Faculty of Literature and Humanities, University of Zabol, Zabol, Iran

## Abstract

**Background:**

Fibrinogen and interleukin-1*β* as a proinflammatory cytokine and interleukin-10 and nesfatin-1 as an anti-inflammatory cytokine have an important role in the development and prevention of systemic inflammation and incidence of obesity-induced diseases. Thus, this study is aimed at the interaction effects of aerobic training and oak husk hydroalcoholic extract consumption on plasma levels of fibrinogen, interleukin-1*β*, nesfatin-1, and interleukin-10 in obese elderly male mice.

**Materials and Methods:**

In this experimental study, 40 fat male mice were fed a high-fat diet for 4 weeks to induce obesity, and subsequently, they were divided randomly into four groups: control, supplement, exercise-placebo, and exercise-supplement. The training groups performed aerobic exercise 5 days a week for 6 weeks (approximately 80-75% VO_max_^2^). The supplement groups received a solution of oak husk hydroalcoholic extract at a dose of 20 milligram per kilogram of body weight for 6 weeks. Blood samples were taken 48 h after the last training session, and the levels of IL-10, fibrinogen, IL-1*β*, and nesfatin-1 were measured. Data were analyzed using one-way ANOVA and LSD post hoc tests.

**Results:**

The results showed that six-week training and oak husk hydroalcoholic extract consumption significantly increased the levels of IL-10 and nesfatin-1 in experimental groups (*P* < 0.001). Also, the levels of fibrinogen and IL-1*β* decreased significantly in training groups. Averages between group variations of all indicators were statistically significant, and they were more meaningfully pronounced in the exercise-supplement group than other groups (*P* ≤ 0.05).

**Conclusions:**

Considering the results of the present study, the use of moderate aerobic exercise and oak husk hydroalcoholic extract is recommended to reduce the risk of obesity; it may also have a positive effect on inflammatory factors.

## 1. Introduction

The world's elderly population is growing, especially in developing countries. Among the issues that elderly people face include obesity and its related cardiovascular diseases [1]. There are increasing studies on the prevalence and prevention of obesity and its related diseases and all emphasize on regulating appetite and energy metabolism [1, 2]. On the other hand, exercise is considered a possible effective factor in controlling inflammation induced by obesity [2]. Findings suggest that an increase in some inflammatory factors such as fibrinogen and interleukin-1-beta (IL-1*β*) may be associated with the development and progression of obesity, and regular exercise can lower the cardiovascular risk factors and consequently reduce mortality rate by reducing inflammatory indicators and body weight [3].

Exercise seems to reduce the long-term inflammation associated with obesity induced by a high-fat diet [4]. Leptin, adiponectin, ghrelin, interleukin-6, and insulin have important effects on the regulation of the hormonal response to long-term and short-term exercise. These mediators also respond to different physical activities and are interrelated in some aspects. Exercise pressure is a factor that links these hormonal mediators, energy balance, and certain interactions between them [4, 5]. One suggested way to analyze this is the relationship between fibrinogen, IL-1*β*, interleukin 10 (IL-10), and nesfatin-1. Fibrinogen is one of the indicators of general inflammation and predicts cardiovascular disease [6]. IL-10, on the other hand, is an anti-inflammatory cytokine that protects obese individuals against the overinflammatory response that is more common in them [4]; nesfatin-1 is also an adipokine that plays a role in the regulation of appetite and metabolism, especially in reducing food intake and anorexia [7].

Several studies have shown the anti-inflammatory effect of physical activity by increasing anti-inflammatory markers such as IL-10 and nesfatin-1 and decreasing proinflammatory markers such as fibrinogen and IL-1*β*-like [4–6, 8]. For example, in 2013, Goebel-Stengel and Wang showed that factors such as exercise directly affect hypothalamic neurons involved in regulating food intake and energy metabolism through IL-10- and nesfatin-1-mediated actions [9]. In 2013, Haghshenas et al. showed that 12 weeks of resistance training significantly increased plasma levels of nesfatin-1 and IL-10 [10]. In 2019, the results of Ghasemi and Nayebifar also showed a significant decrease in serum fibrinogen in overweight women after 10 weeks of interval training along with a green tea supplementation [6].

Recently, the use of medicinal plants as nutritional interventions has been considered along with exercise programs by researchers due to its naturalness and having no side effects. Among them, oak with its scientific name *Quercus*, is among the plants that have many applications in traditional medicine. Studies have shown that the consumption of the hydroalcoholic extract of oak husks with antioxidants, such as tannins, gallic acid, malic acid, and quercine, increases the protective role in heart patients and reduces inflammation induced by obesity. The results of the study of Ahmadvand et al. in 2012 confirmed the inhibitory effect of the hydroalcoholic extract of oak husks on the oxidation of blood lipids by reducing inflammatory cytokines [11]. Also, in 2010, Rivas-Arreola et al. reported the protective role of oak leaf extract in heart patients [12].

Understanding the effects of oak husk extract and aerobic exercise on body mass loss and inflammatory factors will infuse the intricacy of more efficient physical training and supplementation interventions for control of obesity disorder, since aerobic exercise is frequently suggested as an efficiency protocol. Because of the role of inflammation increase in the pathological development of obesity-related diseases (especially in the elderly) on one hand and the anti-inflammatory effects of regular physical exercise and oak husk extract on weight control and obesity on the other, and due to the fact that few studies have been conducted on this plant extract and the same few studies have evaluated the effect of leaf or fruit extract independently and also no study has simultaneously evaluated the effect of aerobic exercise and consumption of the hydroalcoholic extract of oak husks in obese people, the aim of this study was to evaluate the simultaneous effect of 6 weeks of aerobic exercise with the use of the hydroalcoholic extract of oak husks on plasma levels of fibrinogen, IL-1*β*, IL-10, and nesfatin-1 in obese elderly male mice. The authors hypothesize that oak supplementation along with aerobic exercise may have good effects on improving inflammatory markers in obese elderly male mice.

## 2. Research Method

### 2.1. Statistical Population

The present study was experimental and had a multigroup posttest design with a control group. In this study, 40 male mice (20 months old) with an average weight of 160 ± 10 g were purchased from the RahaAvaranNovinEmertat Company and transferred to a laboratory and kept based on the instructions for laboratory animal care and use (*Principles of Laboratory Animal Care*; NIH Publishing, No. 2-863, revised 1996). In this study, mice were fed for one month a high-fat diet (pellet food for mice with a high-fat diet, made by RahaAvaranNovinEmertat) containing fat with 50% of total energy, 20% protein, and 30% carbohydrates, and animal obesity was confirmed by the Lee index [13]. Then, after matching, the samples were randomly divided into four groups of 10, including the control, supplement, aerobic exercise, and exercise-supplement groups, and were kept under standard conditions with a 12 : 12 light : dark cycle at an average temperature of 22°C and humidity of 10-20%.

### 2.2. Research Protocol

After the mice became obese and groups were formed, the mice in the aerobic exercise group and the supplement-exercise group experienced 6 weeks of aerobic exercise (specific treadmill for mice) from 9 to 11 in the morning, 5 days a week. The training program consisted of 3 stages. In the first stage, called the “acquisition phase,” mice walked on a treadmill in the first week, at a speed of 5 to 10 m/min for 10 min at a 0° slope. After the acquisition phase, the exercises entered the second phase called the “overload stage” which lasted 3 weeks; the speed and duration of training increased in different sessions until they reached the final level (speed of 20 m/min, a 5% slope, and a duration of 40 min). In the third stage, called the “stabilizing phase,” the training continued for 2 weeks at a speed of 20 m/min, a 5% slope, and a duration of 40 min. This intensity of exercise was considered approximately 75-75% of maximal oxygen consumption for obese mice [14]. Of the total training time (40 min), 5 min was for the warm-up at the beginning of each session (speed 10 m/min and 0° slope). Moreover, at the end of each session, 5 min was spent for cooling down, by reducing the speed of the treadmill.

Mice in the control and supplement groups were transported to the training room each day and placed on a treadmill for a period of time similar to the training groups, but they experienced no running. Also, to minimize the effects of the noise of the treadmill, the target groups were placed in the vicinity of a treadmill that was turned on.

### 2.3. Preparation and Consumption of Hydroalcoholic Extract of Oak Husks

To prepare the hydroalcoholic extract of oak husks, oak was collected in Mangesht Heights in Baghmalek City and then green husk was removed from the fruit. The removed green husk of the oak was dried in the shade, without direct sunlight, and then ground to its finest with an electric grinder; 400 g of the powdered husk was then poured into a beaker along with 90% ethanol and kept in a dark place for 72 hours (ambient temperature: 25°C). The extract was then filtered using a vacuum pump, and the solvent was removed by a rotary evaporator at 60 rpm, at 43°C for 4 hours. In order to obtain a dry extract, 1 g of the obtained extract was placed in an oven at 45°C for 24 hours. After the solvent evaporated, the dry matter was weighed with a scale (verification scale value: 0.001 g) and 0.33 g of dry and pure extract was obtained. Using oral gavage, 5 mg/kg of the hydroalcoholic extract of oak husks suspended in autoclaved water was given 5 days per week to mice in the supplement groups. In order to relieve the gavage stress, mice in the control and exercise groups were gavaged 200 *μ*l of autoclaved water daily.

In all stages of the study, animals were provided free access to water, and when mice became obese, the high-fat diet was changed to a standard diet (made by the RahaAvaranNovinAmratat Company; the diet contained a certain combination of different nutrients needed by the animal); based on weekly weighing with standard scales, each day a 10 g normal diet, per 100 g of body weight, was placed in each cage.

### 2.4. Laboratory Methods

48 hours after the last aerobic training and supplementation session and its following 12 hours of fasting, mice were anesthetized by inhaling ether solution in a glass chamber. Then, by cutting the skin in the abdomen and chest, and through opening the abdominal cavities, about 10 ml of blood is taken directly from the heart of the mice with a syringe coated with an anticoagulant (ethylene diamine tetraacetic acid (EDTA)) and was transferred to a test tube that contained EDTA. The collected samples were then centrifuged at a speed of 3000 rpm for 10 min, and the resulting plasma was poured into 1 ml microtubes and transferred to the laboratory to be kept at a temperature of -70°C. After collecting the samples in the posttest phase, all blood samples were taken out of the freezer in one day and the obtained plasma was used to measure the levels of IL-1*β*, IL-10, nesfatin-1, and fibrinogen. In order to measure nesfatin-1, IL-10, and IL-1*β*, the nesfatin-1, IL-10, and IL-1*β* kit specific for mice was used, which is made by the Chinese company Kazabaya, and it has a sensitivity of 3.9 and 1 pg/ml to measure them, respectively. Also, the Clause method [15] was used in order to measure fibrinogen.

### 2.5. Statistical Methods

All data are described according to the mean ± standard deviation. After ensuring the normality of the distribution of research variables using the Shapiro-Wilk test and confirming the assumption of homogeneity of variances with the Leven test, one-way analysis of variance and LSD post hoc tests were used to determine intergroup differences. All calculations and statistical analyses were performed using SPSS software (version 18) with *P* < 0.05.

## 3. Results

**Figure 1:**
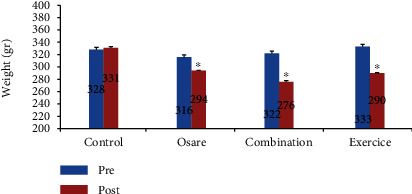
Comparison of weight between groups. ^∗^Significant difference before intervention, *P* < 0.05.

According to the Kolmogorov-Smirnov test, the data have a normal distribution. The results of this study showed that after training and supplementation, the weight of mice in all three groups had a significant decrease compared to the control group and the mice in the exercise-supplement group experienced the highest reduction (*P* ≤ 0.05) (Figure 1).

**Figure 2:**
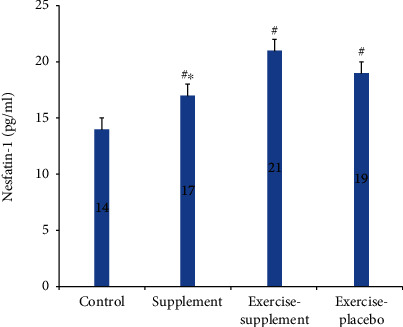
Comparison of nesfatin-1 between groups. ^#^Significant difference with the control group, *P* < 0.05. ^∗^Significant difference with the exercise-supplement group, *P* < 0.05.

**Figure 3:**
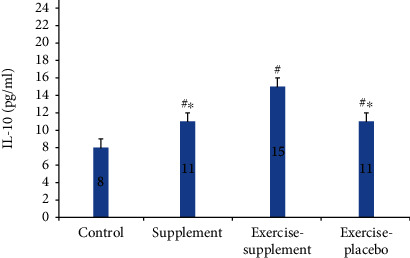
Comparison of IL-10 between groups. ^#^Significant difference with the control group, *P* < 0.05. ^∗^Significant difference with the exercise-supplement group, *P* < 0.05.

Results of one-way ANOVA also revealed significant differences in nesfatin-1, IL-10, fibrinogen, and IL-1*β* (*P* ≤ 0.0001) changes. A post hoc LSD test showed that in the exercise-supplement group, mean changes of nesfatin-1 (*P* ≤ 0.01, *P* ≤ 0.02, and *P* ≤ 0.02, respectively) and IL-10 (*P* ≤ 0.01, *P* ≤ 0.001, and *P* ≤ 0.001) were significantly higher compared to the supplement, exercise-placebo, and control groups (Figures 2 and 3).

**Figure 4:**
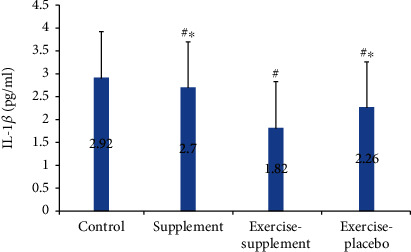
Comparison of IL-1*β* between groups. ^#^Significant difference with the control group, *P* < 0.05. ^∗^Significant difference with the exercise-supplement group, *P* < 0.05.

**Figure 5:**
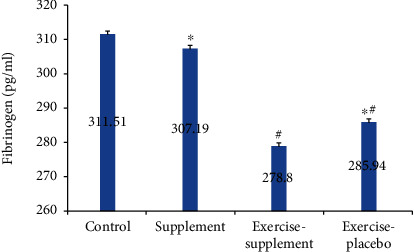
Comparison of fibrinogen between groups. ^#^Significant difference with the control group, *P* < 0.05. ^∗^Significant difference with the exercise-supplement group, *P* < 0.05.

Also, the levels of fibrinogen (*P* ≤ 0.001, *P* ≤ 0.002, and *P* ≤ 0.01, respectively) and IL-1*β* (*P* ≤ 0.03, *P* ≤ 0.01, and *P* ≤ 0.001) were significantly reduced in the exercise-supplement group compared to the supplement, exercise-placebo, and control groups (Figures 4 and 5).

## 4. Discussion

The results of the present study showed that 6 weeks of aerobic exercise and supplementation with hydroalcoholic extract of oak husks significantly reduced IL-1*β* and fibrinogen levels and significantly increased IL-10 and nesfatin-1 levels. The anti-inflammatory effect of exercise has recently been studied, and contradictory results have been obtained. In general, most research reports show that regular physical activity improves the level of inflammatory cytokines [2, 4, 6]. In this regard, Speretta et al. reported in 2012 an increase in IL-10 and a decrease in IL-1*β* levels in visceral adipose tissue of mice following a period of swimming exercise [16]. In 2013, Gomes da Silva et al. showed that IL-10 levels increased and IL-1*β* and fibrinogen levels decreased after regular aerobic exercise in elderly mice [17]. In 2017, Pedersen et al. showed similar results in studying the effect of regular exercise on cardiometabolic patients [18]. These researchers believe that one reason for an increase in IL-10 and a decrease in IL-1*β* levels after exercise is the increase in fat oxidation, and as a result, a reduction of adipose tissue, including visceral fat, as well as the reduction of inflammation. It seems that a decrease in fat mass along with a decrease in the penetration of macrophages into adipose tissue and the conversion of macrophage monocytes (M1) to the macrophage phenotype (M2), causes an increase in anti-inflammatory cytokines such as IL-10 and a decrease in proinflammatory cytokines such as IL-1*β* [19]. On the other hand, with regard to the possibility of a decrease in the levels of fibrinogen synthesized by liver cells, we can mention mechanisms in the musculoskeletal system in relation to regular exercise, in which the activity of cytokines such as IL-1*β* may decrease [6]. Research has also shown that IL-1*β* responses decrease with increasing fitness level [5], so it is likely that IL-1*β* decreases after a period of regular exercise, which in turn can lead to a decrease in fibrinogen synthesized by the liver [5, 6].

Despite this, some studies have reported inconsistent results. For instance, Bartlett et al. in their 2018 study of the effect of 10 weeks of intense intermittent aerobic exercise on elderly patients with osteoarthritis showed that regular exercise, despite its resultant weight loss and reduction of fat percentage and an improvement of the innate immune cell function and the cardiorespiratory system, had no significant effect on the markers of IL-1*β*, IL-10, and CRP [20]. The reason for the inconsistency of the results can be attributed to varying diseases, the resting levels of inflammatory markers, and the training protocol. It seems that the amount of change in inflammatory variables usually depends on their baseline level, and that significant changes resulting from exercise are more substantial and noticeable in people with higher baseline levels of these variables. Participants in the present study were also obese nonathletes, and since cytokine levels are associated with obesity [4] and are higher in obese individuals, the rates of change in these markers have significantly decreased after exercise intervention. In 2012, Stensvold et al. did not report any significant changes in serum levels of IL-6, IL-1*β*, and body composition after 31 months of endurance exercise (with an intensity of 80-90% VO_max_^2^) on 31 sedentary individuals with metabolic syndrome [21]. It seems that the reason for the discrepancy between the results of this study and the findings of the present study can probably be attributed to the high intensity of exercise and the fact that there is no change in body composition after 12 weeks of exercise. In addition, it seems that the type of exercise can affect the rate of change. Exercise training programs with different intensities and durations cause various changes in cytokine levels [20, 21]. There are contradictory findings regarding the appropriate intensity of exercise that causes a reduction inflammatory factors and noticeable anti-inflammatory effects of physical activity [6, 18]. In exercise training programs that have moderate or high intensity and duration, inflammatory variables are affected more and hence they cause a reduction in inflammatory factors; conversely, exercise training programs with lower intensities have less effect on inflammatory factors.

In the present study, significant changes in fibrinogen and IL-1*β* markers were observed only in the training groups. The reason that there is no change in these two markers in the supplement group is probably, on one hand, an insufficient dose of supplement, and on the other hand, the very strong impact that anti-inflammatory supplements have on these two markers. It can also be inferred that exercise is probably a more effective factor than supplement alone in regard to the two markers. In addition, it can be said that the small number of samples in the present study is probably one of the main reasons that these markers in the supplement group are not significant. However, in the future, more studies should be conducted analyzing more samples to evaluate the effect of the hydroalcoholic extract of the oak husk supplement.

The findings of the present study also showed that 6 weeks of exercise increased the level of nesfatin-1. Consistent with the findings of the present study, in 2013, Haghshenas et al. showed that 12 weeks of endurance exercise significantly increased plasma levels of nesfatin-1 and IL-10 [10]. Nevertheless, the same researchers noted a significant reduction of nesfatin-1 in a 2014 study examining the effect of 8 weeks of endurance exercise with a high-fat diet on young obese mice. Among the possible explanations for discrepancies, we can note the age and diet of subjects. In Haghshenas et al.'s study, the analysis was performed on young mice that were on a high-fat diet [22].

As mentioned, because it produces oxidative stress and causes inflammatory conditions, obesity is one of the factors that increases the basal levels of IL-6, IL-1*β*, and fibrinogen and reduces anti-inflammatory markers such as IL-10 and nesfatin-1. Reducing weight improves the concentration of these markers in serum since it reduces oxidative stress and inflammation. Adipose tissue volume is undoubtedly most associated with circulating inflammatory markers. Low levels of inflammation in more active individuals may be mainly due to a low absolute amount of total fat and visceral fat [2, 6]. In 2015, Mifune et al. attributed the weight loss that comes with regular exercise to changes in the beta-oxidation of fat stored in adipocytes [23]. In the present study, the weight variable decreased significantly after 6 weeks of exercise. It is possible that this improvement in body composition could be bilaterally associated with low levels of inflammatory cytokines and increased anti-inflammatory markers.

Also, the findings of the present study showed that daily consumption of 20 mg/kg of the hydroalcoholic extract of oak husk for 6 weeks caused a significant increase in nesfatin-1 and IL-10 levels and weight loss. Although little research has been done on the hydroalcoholic extract of oak husk, other studies have shown the protective role of oak leaf extract in heart disease as well as the antibacterial characteristic of oak leaf and bark extract [12]. In the present study, the combined effect of aerobic exercise and the hydroalcoholic extract of oak husk was investigated and it was found that after 6 weeks of aerobic exercise with supplements, the weight of the subjects and subsequently their IL-1*β* and fibrinogen markers decreased and nesfatin-1 and IL-10 levels increased. Moreover, in the present study, since these markers in the exercise-supplement group experienced more changes than the other groups, we can point to the role of the hydroalcoholic extract of oak husk as a strong anti-inflammatory supplement along with aerobic exercise in controlling obesity. According to the researcher of the present study, so far there is no study that simultaneously examines the effects of taking this supplement and aerobic exercise on inflammatory factors in obese elderly people, and the present study is the first in this regard. The polyphenolic and tannin compounds in the hydroalcoholic extract of oak husk, on the one hand, reduce the secretion of cytokines such as IL-1*β*, owing to their inhibitory effect on macrophages, and on the other hand, increase the levels of nesfatin-1 and IL-10 [11, 12]. In addition, if taking this supplement is combined with regular physical training, this increase will probably be more tangible.

Findings indicate that regular aerobic exercise has anti-inflammatory effects and suppresses low-grade systemic inflammation [24]. Research suggests that exercise may help reduce inflammation by modulating adipokines associated with insulin resistance secreted by adipose tissue [25]. Following exercise, IL-6 circulating levels increase and stimulate the IL-10 anti-inflammatory cytokine, and finally, inflammatory cytokines may act as a response to prevent obesity by modulating both energy intake and consumption [24, 25]. In addition, it appears that inflammatory and anti-inflammatory cytokines may act as a link between peripheral tissues and the central nervous system in controlling energy balance, and perhaps a reduction in accumulated energy resulting from an increase in physical activity, followed by controlling long-term inflammation caused by obesity, applies to both obese and nonobese conditions. It is generally believed that exercise plays a role in controlling and modulating inflammation through three major mechanisms: reducing visceral fat, increasing the production of anti-inflammatory cytokines, and reducing inflammatory cytokines [26].

One of the limitations of the present study is the small number of samples and the impossibility of research due to few studies on the hydroalcoholic extract of the oak husk supplement. Also, due to financial constraints, it was not possible to measure more indicators for more accurate conclusions.

## 5. Conclusion

In general, from the results of this study, it can be concluded that regular aerobic exercise (for 6 weeks, 5 days a week, with 75% intensity of maximum oxygen consumption) along with taking the hydroalcoholic extract of oak husk as a supplement (5 days-20 mg/kg per week) plays an important role in prompting positive changes in regard to reducing inflammation and weight in obese elderly mice. The synergistic effects of the supplement and exercise significantly increase nesfatin-1 and IL-10 levels and significantly decrease IL-1*β* and fibrinogen levels compared to when exercise or supplement is used alone.

## Data Availability

Data are presented within the article in the form of tables, text, and figures. Other data will be made available upon request.

## Additional Points

*Research Involving Human Participants and/or Animals*. Research involves animal participants.
